# Minoxidil Promotes Hair Growth in a Mouse Model of Telogen Effluvium Induced by Lipopolysaccharide

**DOI:** 10.1111/1346-8138.70062

**Published:** 2025-11-16

**Authors:** Yuji Tani, Shiro Makihara, Akihisa Morito, Manabu Ohyama

**Affiliations:** ^1^ Pharmacology, Self‐Medication Research Center, Research Headquarters Taisho Pharmaceutical Co., Ltd. Tokyo Japan; ^2^ Department of Dermatology Kyorin University School of Medicine Tokyo Japan

**Keywords:** alopecia, cytokines, lipopolysaccharides, mice, minoxidil

## Abstract

Telogen effluvium (TE) refers to a transient hair loss condition due to the global and premature transition of scalp hair follicles from the anagen to the telogen phase of the hair cycle leading to excessive hair loss, which is triggered by a variety of physical/emotional stress, including severe infectious diseases. Because of its self‐healing nature, attempts to develop remedies for TE have rarely been conducted; still the demand for TE treatment exists as the condition can significantly impair the quality of life of affected individuals. This study aims to establish a mouse model of TE induced by lipopolysaccharide (LPS) and evaluate the therapeutic effects of topical minoxidil (tMXD), an established hair growth‐promoting medication. Mice had their dorsal hairs plucked to induce anagen transition and were administered LPS. LPS elicited a condition mimicking a cytokine storm with increases in interleukin‐6 levels and neutrophil counts. Accelerated transition of hair follicles into telogen was observed in LPS‐treated mice. Intriguingly, tMXD accelerated hair regrowth in LPS‐treated mice, resulting in earlier achievement of complete hair regrowth compared to controls. In aggregate, a previously unreported TE mouse model is established, and its use supports the ameliorative effect of tMXD on TE.

## 1 Introduction

Telogen effluvium (TE) is a condition characterized by excessive hair loss attributed to physical/emotional stress, such as childbirth, febrile diseases, major surgery, medication, and nutritional deficiencies [1]. Recently, TE has been noted as a major cause of hair loss as a sequela of COVID‐19 [2]. Despite the self‐healing nature of TE, the demand for effective treatments still exists; massive and progressive hair loss can significantly impair the quality of life of affected individuals potentially leading to mental distress and difficulties in social life [1]. To date, attempts to develop remedies for TE have rarely been conducted. Designing a clinical study can be challenging as spontaneous recovery is expected. The development of TE animal models is also technically challenging, as the physiological characteristics of animal (mostly murine) hair follicles (HFs), including the hair cycle, are distinct from those of humans. In this study, we attempted to establish a novel TE mouse model, by systemic lipopolysaccharide (LPS) administration, which is expected to trigger a cytokine storm [3, 4] and subsequently cause TE. Taking advantage of digital analysis to measure skin brightness reflecting the hair cycle status, the therapeutic effects of topical minoxidil (tMXD) [5] on the TE model mouse were investigated.

## 2 Methods

C57BL/6N mice had their dorsal hair plucked. Ten days postplucking, LPS derived from *Escherichia coli* O55:B5 was dissolved in saline and administered intraperitoneally (i.p.). Body weight was periodically measured. The day after LPS administration, blood was collected from the tail vein. Leukocyte classification was performed using blood smear specimens stained by the May‐Grünwald‐Giemsa method. Plasma interleukin (IL)‐6 levels were measured by enzyme‐linked immunosorbent assay. Seven days post‐LPS administration, the transition from anagen to telogen was evaluated. Newly grown hairs in the plucked dorsal area were shaved, and the dorsal skin brightness (*L* value) using a colorimeter [6], and dermoscopic findings were recorded.

Skin samples were collected and processed to generate hematoxylin‐and‐eosin‐stained sections. Hair cycle score (HCS) was subsequently determined by assessing histologically detectable hair cycles of HFs. HCS was calculated by assigning numerical values to each hair cycle stage (anagen VI and catagen I = 100, catagen II = 200, catagen III = 300, etc.) [7]. In another set of mice, the dorsal skin was shaved 25 days after plucking and 5% MXD lotion composed of ethanol, water, and 1,3‐butylene glycol as the major ingredient was topically applied five times a week. The shaved area was monitored and recorded at 10% increments.

Statistical analyses were performed using Dunnett's or Tukey's test for parametric data, and Steel's or Steel‐Dwass's test for nonparametric data.

## 3 Results

### 3.1 Intraperitoneal LPS Administration Elicited a Condition Mimicking a Cytokine Storm

**FIGURE 1:**
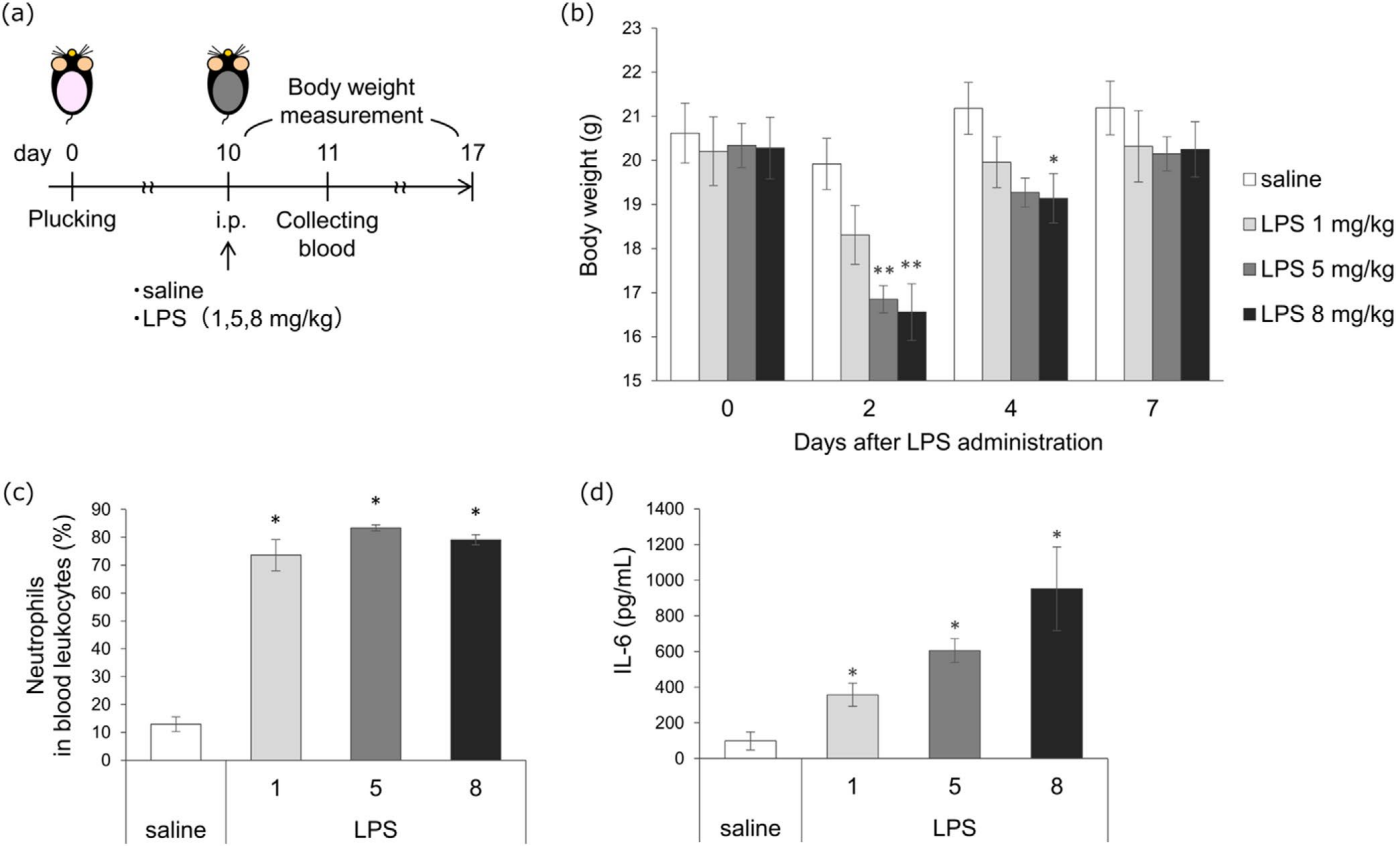
Elicitation of lipopolysaccharide (LPS)‐induced cytokine storm‐like condition in mice. (a) A schematic representation of the experimental procedure. (b) Change in body weight. (c) The percentage of neutrophils in the blood leukocytes. The number of neutrophils was counted in two hundred leukocytes per animal. (d) Plasma interleukin‐6 level. (b–d) *n* = 5. *p < 0.05, **p < 0.01 versus saline. Mean ± SE. (c, d) The *x*‐axis represents the dose of LPS (mg/kg). i.p., intraperitoneal; IL‐6, interleukin‐6; LPS, lipopolysaccharide.

LPS (1, 5, 8 mg/kg) was administered (Figure 1a), resulting in body weight loss 2 days postadministration (saline 19.92 ± 0.58 g vs. LPS 8 mg/kg 16.56 ± 0.64 g; *p* < 0.01), with recovery to control levels by day 7 (Figure 1b). The proportion of neutrophils in the blood exceeded 70% 1 day postadministration (*p* < 0.05) (Figure 1c). Plasma IL‐6 levels significantly increased (saline 98.53 ± 50.83 pg/mL vs. LPS 8 mg/kg 951.76 ± 234.54 pg/mL; *p* < 0.05) (Figure 1d), indicating successful induction of inflammatory cytokine responses.

### 3.2 tMXD Promoted Hair Regrowth After LPS‐Induced Experimental Telogen Transition in Mice

**FIGURE 2:**
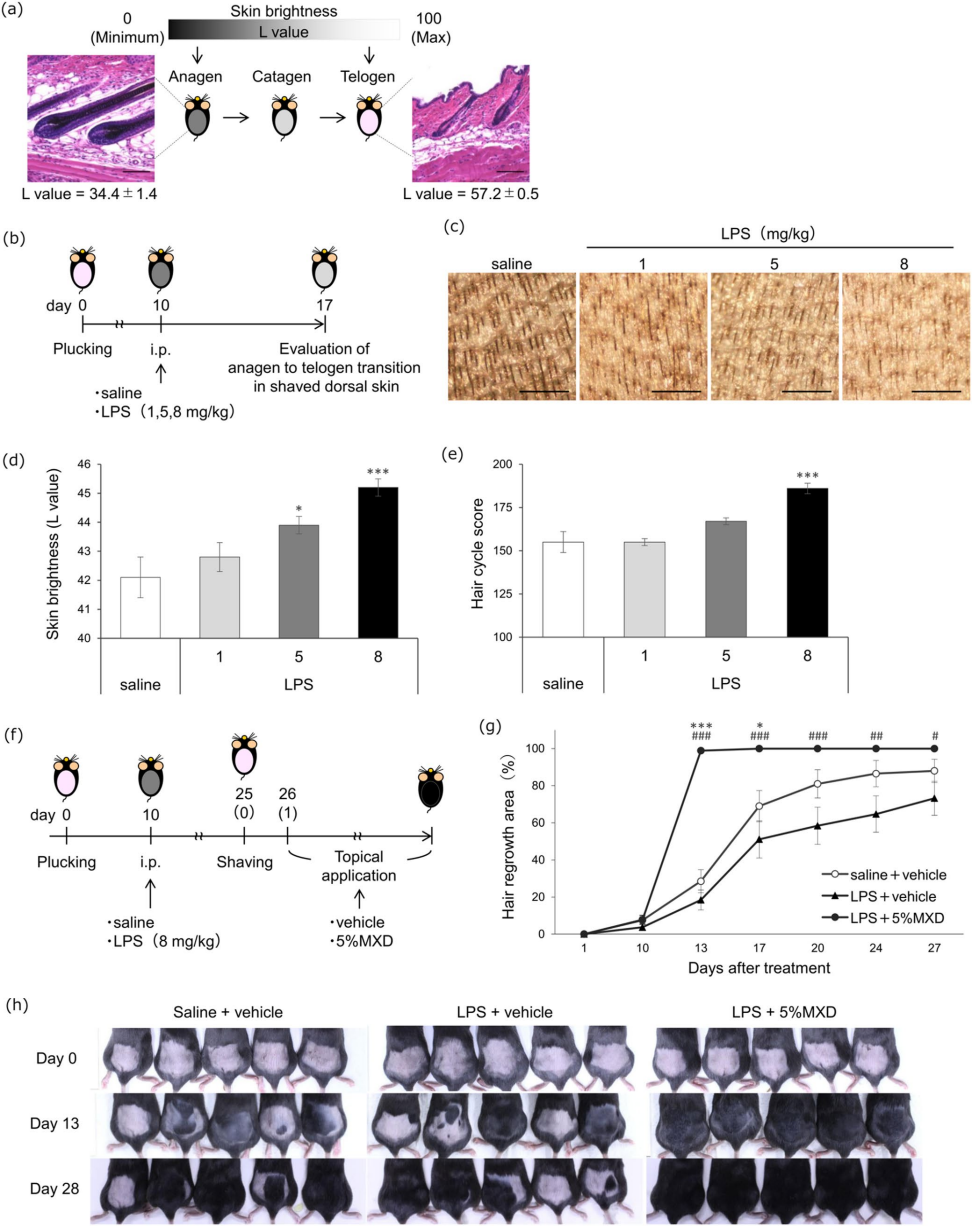
Topical minoxidil (tMXD) accelerated hair cycle transition from telogen to anagen in lipopolysaccharide (LPS)‐induced mouse telogen effluvium model. (a) A schema illustrating the skin brightness (*L* value) used for hair cycle evaluation. Note that *L* values reflect the histopathologically detectable changes in hair cycle phases (The values below the histopathology panels indicate the correlation between the *L* value and the hair cycle). Bars = 100 μm. *n* = 3. (b) A schematic representation of the experimental procedure. (c) Representative dermoscopic images of the shaved dorsal skin of individual mouse groups. Bars = 500 μm. (d) Changes in the *L* value in the shaved dorsal skin. The *x*‐axis represents the dose of LPS (mg/kg). *p < 0.05, ***p < 0.001, versus saline. *n* = 10. (e) Changes in the hair cycle score (HCS) in the shaved dorsal skin. Each mouse with at least 50 individual hair follicles was assessed to a defined hair cycle stage and HCS. The *x*‐axis represents the dose of LPS (mg/kg). ***p < 0.001, versus saline. *n* = 5. (f) A schematic representation of the experimental procedure. The numbers in parentheses indicate days after the start of treatment. (g) Effect of tMXD on hair growth in the mouse TE model. *p < 0.05, ***p < 0.001, versus saline + vehicle; #*p* < 0.05, ##*p* < 0.01, ###*p* < 0.001, versus LPS + vehicle; comparisons performed each day. *n* = 18–20 mice. (h) Representative dorsal photographs acquired on Days 0, 13, and 28 following the initiation of tMXD. (a, d, e, g) Mean ± SE. i.p., intraperitoneal; LPS, lipopolysaccharide; MXD, minoxidil.

C57BL/6 mice show skin brightness changes (*L* value) during the transition from anagen to telogen phase as confirmed by histopathological examination [6, 7] (Figure 2a). After LPS administration, *L* values, dermoscopic findings, and HCS of the shaved dorsal skin were assessed (Figure 2b). Compared to control, the LPS‐treated groups showed fewer hair shafts, higher *L* values (saline 42.1 ± 0.7 vs. LPS 8 mg/kg 45.2 ± 0.3; *p* < 0.001), and greater HCS (saline 155 ± 6 vs. LPS 8 mg/kg 186 ± 3; *p* < 0.001), with the 8 mg/kg LPS group showing the greatest increase in both parameters, indicating an accelerated transition from the anagen to telogen phase (Figure 2c–e).

Using 8 mg/kg LPS‐treated TE model mice, the therapeutic effect of tMXD was evaluated (Figure 2f). Of note, 5% MXD lotion significantly accelerated hair regrowth as detected by the increase in hair regrowth area (day 13: LPS + 5% MXD 98.9% ± 0.8%, saline + vehicle 28.5% ± 6.2%, LPS + vehicle 18.4% ± 5.4%; vs. LPS + 5% MXD, both *p* < 0.001), demonstrating its ameliorative effect (Figure 2g,h).

## 4 Discussion

Body weight loss, tissue neutrophil infiltration, and elevated blood IL‐6 have been used as cytokine‐storm readouts [3, 4]. Of Headington's five TE subtypes, infection‐induced TE is classed as an “immediate anagen release” condition [8]. In this regard, the accelerated transition from anagen to telogen phase in LPS‐treated mice mimicked, at least in part, the pathophysiology of postinfection TE. Desmoglein 3 knockout (Dsg3 KO) mice are known to present with alopecic patches due to telogen hair loss [9]: however, Dsg3 KO mice do not recapitulate the hair cycle abnormality that is the most representative characteristic of TE. Anagen induction by plucking prior to LPS administration is necessary in our model due to the predominance of telogen hair follicles in mice [7] and that excessive hair loss cannot be observed in LPS‐treated mice due to experimentally necessary shaving. Despite that, the TE mouse model developed in this study can provide a tool to examine an ameliorative effect of tested reagents in the TE setting.

Previous studies have evaluated the effects of interferon (IFN)‐gamma, polyinosinic‐polycytidylic acid and the combination of tumor necrosis factor‐alpha, IL‐1beta, and IFN‐gamma, on the hair cycle [10, 11]. These studies focused on local skin pathology including inflammatory and hair cycle change. This study is novel in that more global hair cycle change was evaluated. We are aware that clinicopathological changes reproduced in our TE mouse model are limited. Of note, massive club hair shedding cannot be observed. Genetic or pharmacological modulation to induce exogen simultaneously with telogen is necessary to achieve this in mice, which represents a future step in experimental TE mouse model development.

MXD promotes the anagen phase of the hair cycle [5]. tMXD is available as 1% and 5% formulations in Japan; [12]; chemical‐stimuli could elicit anagen transition; however, the topical vehicle and 5% MXD did not provoke inflammatory changes in mice providing a rationale for selecting 5% tMXD. Earlier transition from the telogen to anagen phase observed in the tMXD group supported the hair cycle modulatory effect of MXD. In line with this, in a mouse model of hair loss induced by sonic stress, in which tMXD inhibited early catagen entry [13].

This study has several limitations. As mentioned above, the pathophysiology of human TE including its trajectory was just partially recapitulated in this TE mouse model. tMXD can potentially cause adverse events best represented by contact dermatitis [14]; however, safety evaluation was not conducted in this study. Further comprehensive analyses and additional clinical trials are essential to substantiate the results.

In aggregate, a previously unreported TE mouse model is established, and its use supports the ameliorative effect of tMXD on TE.

## Ethics Statement

All animal experiments were approved by the Animal Experiment Committee of Taisho Pharmaceutical Co. Ltd. (AN13706, AN14113, AN14580, AN14581).

## Conflicts of Interest

M.O. serves as a medical advisor for Taisho Pharmaceutical Co. and receives an advisory fee for this study. M.O. also receives lecture fees from Eli Lilly Japan K.K. and Pfizer Inc., advisory fees from Eli Lilly Japan K.K., Pfizer Inc., Bristol Myers Squibb K.K., Abbvie G.K., and RHOTO Pharmaceutical Co., and research grants from Maruho Co., Sun Pharma Japan Ltd., ADVANTEST Co., Sanofi K.K., Kyowa Kirin Co. and Shiseido Co. for the projects on hair diseases but not directly related to the current work. Y.T., S.M. and A.M. are paid employees of Taisho Pharmaceutical Co. M.O. is an Editorial Board member of the *Journal of Dermatology* and a coauthor of this article. To minimize bias, they were excluded from all editorial decision‐making related to the acceptance of this article for publication.

## Data Availability

The data that support the findings of this study are available from the corresponding author upon reasonable request.
